# Prevalence and effects on outcomes of intracranial tumors and structural alterations in children with central precocious puberty and early and fast puberty

**DOI:** 10.3389/fendo.2026.1880616

**Published:** 2026-07-24

**Authors:** Xian Wu, Yanhong Li, Huamei Ma, Qiuli Chen, Jun Zhang, Song Guo, Liulu Xie, Minlian Du

**Affiliations:** First Affiliated Hospital of Sun Yat-sen University, Guangzhou, Guangdong, China

**Keywords:** central precocious puberty, early and fast puberty, gonadotropin-releasing hormone agonist, intracranial lesions, magnetic resonance imaging

## Abstract

**Objective:**

To investigate intracranial lesion prevalence in children with central precocious puberty (CPP) and early and fast puberty (EFP), and assess impacts on progression and GnRHa response.

**Methods:**

This retrospective study included 1,087 CPP/EFP patients (2011–2023) who underwent brain MRI. Patients were stratified by MRI findings: no lesion, other (incidental) lesions, and pathogenic lesions (hypothalamic hamartoma/glioma). Clinical, hormonal, bone age, and growth parameters were compared. GnRHa dosing and therapeutic responses were evaluated.

**Results:**

Intracranial lesions were detected in 14.5% of girls and 19.6% of boys (P = 0.089). Detection was age-dependent: children under specific thresholds (girls <6y, boys <7y) had significantly higher rates than older peers (33.7% vs. 12.1%, P <0.001; 53.8% vs. 16.9%, P = 0.004). Pathogenic lesions (n=26) occurred exclusively in CPP, linked to earlier puberty onset (girls: 1.9y; boys: 1.0y) and elevated luteinizing hormone (LH) levels (P<0.05). Among treated girls (n=312), those with pathogenic lesions required higher 6-month GnRHa doses (P = 0.025) and exhibited faster initial growth velocity (P = 0.007), despite similar hormone suppression and 1-year height SDS changes. Incidental lesions (e.g., Rathke’s cysts) did not alter progression or treatment response.

**Conclusion:**

Routine MRI is warranted for younger CPP children (girls <6y, boys <7y) due to high pathogenic lesion rates. Conversely, older CPP and all EFP cases show low pathogenic prevalence, questioning universal MRI screening. While incidental lesions require no management alteration, CPP with hypothalamic hamartoma/glioma presents with earlier onset and heightened axis activation, necessitating higher GnRHa doses.

## 1 Introduction

Central precocious puberty (CPP) is a common pediatric endocrine disorder characterized by the premature activation of the hypothalamic-pituitary-gonadal axis (HPG axis) (1). CPP is classified into idiopathic (ICPP) and organic (OCPP) forms (2). The latter is frequently associated with intracranial lesions, such as hypothalamic hamartomas and gliomas. Brain MRI serves as a critical diagnostic tool for identifying OCPP and excluding organic intracranial etiologies (3). However, controversies persist regarding the actual prevalence of intracranial lesions in children with CPP—particularly across different ages, sexes, and clinical subtypes such as early and fast puberty (EFP)—as well as the clinical significance of different lesion types and their impact on pubertal progression and response to standard therapy with gonadotropin-releasing hormone agonists (GnRHa) (4, 5). While previous studies suggested a higher proportion of organic causes in boys and younger children, recent evidence indicates that these incidence rates may have been overestimated (6–8). Furthermore, it remains unverified whether common “incidental” lesions, such as pituitary Rathke’s cleft cysts and pineal cysts, influence pubertal progression, lead to rapidly progressive CPP, or necessitate supratherapeutic GnRHa doses. Clarifying these issues is essential for optimizing clinical MRI screening strategies and formulating individualized treatment plans. This study aims to characterize the epidemiological features of intracranial lesions in a large cohort of children with CPP and EFP through retrospective analysis and to explore the potential impact of different lesion types on disease progression and GnRHa efficacy.

## 2 Methods

### 2.1 Study population

We retrospectively collected clinical data from children treated at the Child Growth Center of the First Affiliated Hospital of Sun Yat-sen University between January 2011 and June 2023.Inclusion criteria (1): Diagnosis of Central Precocious Puberty (CPP) according to the 2015 “Consensus on Diagnosis and Treatment of Central Precocious Puberty,” or diagnosis of Early and Fast Puberty (EFP) (defined as initiation of hypothalamic-pituitary-gonadal axis activation in girls aged 8–10 years or boys aged 9–11 years, with a ratio of bone age advancement to chronological age progression>1) (9); (2) Availability of complete brain and/or sellar region magnetic resonance imaging (MRI) data.

Exclusion criteria: Cases of peripheral precocious puberty, patients receiving concomitant medications such as growth hormone during follow-up, or those with incomplete medical records.

Not all patients meeting the inclusion criteria received GnRH analogue (GnRHa) therapy. The decision to withhold treatment was based on several clinical and practical considerations: (1) a diagnosis of slowly progressive puberty with a favorable prognosis at initial presentation; (2) advanced bone age indicating limited remaining growth potential; (3) lack of post-treatment follow-up data at our center for some patients; and (4) parental decisions regarding treatment costs and potential side effects.

### 2.2 Study design and grouping

Demographic information, age at pubertal onset, physical examination findings, sex hormone levels (basal and post-GnRH stimulation), pelvic ultrasound/testicular volume data, and bone age (assessed via the Greulich-Pyle atlas method) were collected. Bone age was assessed independently by two experienced pediatric endocrinologists, and any discrepancies were resolved by consensus. All MRI images were re-evaluated by a single dedicated radiologist to ensure consistency. Based on MRI findings, patients were stratified into three groups:

Group A (No Lesion) No intracranial lesions detected.

Group B (Other Lesions) Presence of incidental structura abnormalities, including Rathke’s cleft cysts, pineal cysts, non-suprasellar arachnoid cysts, pituitary microadenomas, or thickened pituitary stalk.

Group C (Pathogenic Lesions) Presence of lesions with established causality for CPP, specifically hypothalamic hamartomas or low-grade gliomas.

### 2.3 Outcome measures

Comparisons among the three groups included:

1. Baseline characteristics at initial diagnosis: age at pubertal onset, bone age (BA), bone age advancement (BA minus chronological age [CA]), height SDS for bone age, and basal/stimulated LH and follicle-stimulating Hormone (FSH) levels.
2. Therapeutic outcomes in the subgroup receiving GnRHa therapy for at least one year: cumulative GnRHa dosage, basal and peak stimulated LH levels at 3-6 months post-treatment, growth velocity (GV) at 6 months, and height SDS for bone age along with its change (ΔHtSDS-BA) after one year of treatment.
3. Treatment Strategy: The initial GnRHa dosage was determined based on baseline characteristics. Patients with pathogenic lesions received a higher cumulative dose due to more severe HPG axis activation at diagnosis (higher peak LH levels), rather than dose escalation during follow-up.

### 2.4 Statistical analysis

Statistical analyses were performed using SPSS version 27.0. Continuous variables with a normal distribution were expressed as mean ± standard deviation (SD) and compared using Analysis of Variance (ANOVA). Non-normally distributed variables were expressed as median (interquartile range [IQR]) and compared using the Kruskal-Wallis H test. Categorical variables were compared using the Chi-square test or Fisher’s exact test. A two-sided P-value<0.05 was considered statistically significant.

## 3 Results

### 3.1 General characteristics and overall prevalence of intracranial lesions

A total of 1,087 pediatric patients were included. Among 908 girls, 132 cases of intracranial lesions were identified (14.5%): of these, 19 cases (2.1%) were confirmed pathogenic lesions, comprising 18 cases of hypothalamic hamartoma (2.0%) and 1 case of low-grade glioma (0.1%). The remaining lesions included 70 cases of Rathke’s cleft cyst (7.7%), 20 cases of pineal cyst (2.2%), 6 cases of non-suprasellar arachnoid cyst (4.5%), 12 cases of microadenoma (1.3%), 4 cases of pituitary stalk thickening (0.4%), and 1 case of traumatic brain injury (0.1%). Among 179 boys, 35 cases of intracranial lesions were detected (19.6%), showing no significant difference compared to girls (p=0.089). Of these, 7 cases (4.0%) were confirmed pathogenic lesions, consisting of 5 cases of hypothalamic hamartoma (2.8%) and 2 cases of low-grade glioma (1.1%). The remaining lesions included 14 cases of Rathke’s cleft cyst (7.8%), 6 cases of pineal cyst (3.4%), 5 cases of non-suprasellar arachnoid cyst (2.8%), 2 cases of non-functioning pituitary microadenoma (1.1%), and 1 case of pituitary stalk thickening (0.6%). Regarding surgical intervention, 4 girls (0.4%) underwent surgery, including 1 case of glioma and 3 cases of hamartoma; 2 boys (1.1%) underwent surgery, both for glioma (**Table 1**).

**Table 1:** Detection rates of intracranial lesions in CPP and EFP.

Group	MRI (-) N (%)	Other lesions N (%)	Pathogenic lesions N (%)	Total MRI (+) N (%)
Girls	776 (85.5)	113 (12.4)	19 (2.1)	132 (14.5)
Boys	144 (80.4)	28 (15.6)	7 (3.9)	35 (19.6)
*p*	0.089	0.245	0.235	0.089

The p-values in **Table 1** represent the differences in the distribution of specific MRI findings between the two sexes.

### 3.2 Association of lesion prevalence with age and diagnostic subtypes

The prevalence of intracranial lesions showed a significant age-dependent trend. Among girls, the detection rate in those <6 years old (33.7%, 34/101) was significantly higher than in those ≥6 years old (12.1%, 98/807); notably, the proportion of pathogenic lesions was markedly higher in the younger group (16.8% vs. 0.2%, P<0.001). Similarly, in boys, the detection rate was significantly higher in those <7 years old (53.8%, 7/13) compared to those ≥7 years old (16.9%, 28/166), with a substantially higher proportion of pathogenic lesions (42.9% vs. 0.6%, P<0.001). All 26 cases of pathogenic lesions (23 hypothalamic hamartomas and 3 low-grade gliomas) were exclusively identified in children with central precocious puberty (CPP); none were detected in children with early and fast puberty (EFP) (**Tables 2**, **3**).

**Table 2:** Detection rates of intracranial lesions in girls with CPP/EFP by age group.

Age group	MRI(+) N (%)	Pathogenic lesions N (%)	P-value
MRI (+)	34/101 (33.7)	98/807 (12.1)	<0.001
pathogenic Lesions	17/101 (16.8)	2/807 (0.2)	<0.001

**Table 3:** Detection rates of intracranial lesions in boys with CPP/EFP by age group.

Age group	MRI(+) N (%)	Pathogenic lesions N (%)	P-value
MRI (+)	7/13 (53.8)	28/166 (16.9)	0.004
pathogenic Lesions	6/13 (42.9)	1/166 (0.6)	<0.001

### 3.3 Comparison of baseline clinical characteristics in CPP patients by lesion type

Comparisons among the three groups revealed that the group with pathogenic lesions had a significantly earlier onset of puberty (mean age: 1.9 years for girls and 1.0 year for boys) and higher baseline LH levels as well as higher peak LH levels after stimulation. However, no statistically significant differences were observed among the three groups in the difference between bone age and chronological age at initial diagnosis or in height SDS for bone age (all p>0.05). Furthermore, comparisons between the group with no detected lesions and the group with other lesions showed no statistically significant differences in pubertal onset age, baseline LH and FSH levels, peak stimulated LH levels, the difference between bone age and chronological age, or height SDS for bone age (all p>0.05) (**Tables 4**, **5**).

**Table 4:** Comparison of baseline clinical characteristics in girls with CPP according to lesion type (N = 668).

Characteristic		MRI (-)	Other lesions	Pathogenic lesions	P-value
Age at diagnosis (yrs)		8.2 (7.5, 9.0)	8.1 (7.2, 9.2)	3.2 (1.6, 3.8)*#	<.001
Age at onset of symptoms (yrs)		7.3 (6.7, 7.7)	7.3 (6.3, 7.7)	1.9 (0.7, 3.5)*#	<.001
Height-SDS		0.6 (-0.1, 1.3)	0.6 (-0.1, 1.4)	1.3 (0.4, 2.3)	0.106
BMI-SDS		0.9 ± 1.2	0.7 ± 1.1	1.0 ± 1.4	0.302
BA-CA (yrs)		2.5 (1.9, 3.0)	2.3 (1.5, 3.2)	2.0 (0.7, 3.2)	0.308
Height-SDS for bone age		-1.8 (-2.3, -1.2)	-1.8 (-2.4, -1.2)	-0.5 (-3.4, 1.1)	0.258
Tanner stage	B	3.0 (3.0, 4.0)	3.0 (3.0, 4.0)	3.0 (3.0, 4.0)	0.459
	PH	1.0 (1.0, 2.0)	1.0 (1.0, 3.0)	1.0 (1.0, 2.0)	0.811
Basal LH (IU/L)		1.1 (0.4, 2.9)	0.8 (0.4, 3.1)	3.1 (1.8, 5.0)*#	0.005
Basal FSH (IU/L)		3.9 (2.5, 5.3)	4.0 (2.6, 5.8)	5.1 (4.1, 6.4)	0.079
Peak LH (IU/L)		16.0 (8.4, 31.1)	11.4 (7.7, 24.6)	29.2 (20.7, 51.5)*#	0.005
Uterine body length (mm)		25.0 (21.0, 30.0)	25.0 (21.0, 30.0)	22.5 (19.0, 26.3)	0.261
Left ovarian volume (ml)		1.6 (1.1, 2.0)	1.5 (1.1, 2.0)	1.3 (0.8, 1.9)	0.392
Right ovarian volume (ml)		1.5 (1.2, 2.1)	1.6 (1.2, 2.2)	1.5 (0.8, 1.8)	0.450

B: Tanner stage of breast development (in girls); PH: Tanner stage of pubic hair.

*Significant difference vs. No lesion group; # Significant difference vs. Other lesion group.

Normally distributed data are presented as mean ± SD; non-normally distributed data are presented as median (P25, P75).

**Table 5:** Comparison of baseline clinical characteristics in boys with CPP according to lesion type (N = 89).

Characteristic		MRI (-)	Other lesions	Pathogenic lesions	P-value
Age at diagnosis (years)		9.9 (9.0, 10.5)	9.9 (8.8, 10.4)	1.9 (1.5, 4.4)*#	0.005
Age at onset of symptoms (yrs)		8.7 (8.0, 8.8)	8.3 (7.4, 8.8)	1.0 (0.3, 4.3)*#	<.001
Height-SDS		0.5 (-0.1, 1.5)	1.3 (0.4, 2.0)	2.6 (1.5, 3.0)*	<.001
BMI-SDS		0.5 ± 1.1	0.8 ± 1.6	0.6 ± 1.1	0.820
BA-CA (yrs)		2.5 (1.8, 3.3)	2.9 (2.1, 3.4)	3.1 (1.7, 3.5)	0.532
Height-SDS for bone age		-1.7 (-2.2, -0.9)	-1.4 (-1.8, -0.9)	1.5 (0.0, 3.0)*	0.002
Tanner stage	G	3.0 (2.5, 4.0)	3.0 (3.0, 4.0)	3.0 (2.0, 4.0)	0.511
	PH	2.0 (1.0, 4.0)	3.0 (2.0, 3.0)	3.0 (2.0, 3.0)	0.199
Basal LH (IU/L)		1.6 (1.0, 2.6)	1.9 (1.3, 3.4)	3.7 (3.6, 5.0)*	0.015
Basal FSH (IU/L)		3.2 (2.2, 4.0)	3.1 (2.0, 4.3)	2.2 (1.8, 4.2)	0.735
Peak LH (IU/L)		18.0 (12.4, 26.2)	22.6 (16.4, 28.7)	34.0 (26.6, 40.1)*	0.008

G: Tanner stage of male external genitalia; PH: Tanner stage of pubic hair.

*Significant difference vs. No lesion group; # Significant difference vs. Other lesion group.

Normally distributed data are presented as mean ± SD; non-normally distributed data are presented as median (P25, P75).

### 3.4 Impact of intracranial lesion types on GnRHa therapeutic response

A total of 383 patients (312 girls and 71 boys) received GnRHa therapy and completed a 1-year follow-up. Statistical analysis revealed no significant differences between the CPP and EFP groups in cumulative GnRHa dosage, post-treatment hormone levels, or bone age progression (all p>0.05); therefore, data from these two groups were pooled for analysis. Compared with CPP and EFP patients detected with other intracranial lesions, those with pathogenic lesions required a higher cumulative dose of GnRHa. Furthermore, the growth velocity (GV) remained relatively rapid during the first 6 months of treatment in this group. However, no statistically significant differences were observed in serum baseline LH and FSH levels at 3-6 months post-treatment, peak stimulated LH levels, or the change in height SDS for bone age after 1 year of treatment (all p>0.05). Comparisons between patients with no detected intracranial lesions and those with other lesions showed no statistically significant differences in cumulative GnRHa dosage, serum baseline LH and peak stimulated LH levels at 3-6 months, GV during the first 6 months post-treatment, or the change in height SDS for bone age after 1 year (all p>0.05). Due to absence of post-treatment data for male patients with pathogenic lesions, multi-group comparisons were not performed; however, no differences in efficacy indicators were found between the no-lesion group and the other-lesion group (**Tables 6**, **7**).

**Table 6:** Clinical characteristics of girls with CPP and EFP after GnRHa treatment (N = 312).

Characteristic	MRI (-)	Other lesions	Pathogenic lesions	P-value
Tanner stage	3.2 ± 0.7	3.5 ± 0.7	3.0 ± 0.9	0.080
BA-CA (yrs)	2.4 (1.8, 2.9)	2.6 (1.6, 3.2)	1.1 (0.1, 4.2)	0.362
Height-SDS for bone age	-1.8 (-2.3, -1.4)	-1.9 (-2.2, -1.3)	-0.3 (-2.5, 1.8)	0.074
Uterine body length (mm)	24.9 ± 6.2	27.2 ± 8.0	22.6 ± 4.1	0.077
cumulative 6-month therapeutic dose (μg/kg)	682.2 (585.3, 750.8)	669.1 (594.2, 739.5)	852.0 (649.4, 1143.6)*#	0.025
Basal LH (IU/L) (3-6m)	0.2 (0.1, 0.4)	0.2 (0.1, 0.4)	0.3 (0.2, 0.4)	0.230
Peak LH (IU/L) (3-6m)	0.7 (0.4, 1.1)	0.7 (0.4, 1.0)	0.8 (0.7, 1.1)	0.602
Uterine body length (mm) (6m)	20.3 ± 4.3	20.7 ± 4.2	17.3 ± 1.9	0.151
Tanner stage (6m)	2.8 ± 0.9	3.3 ± 0.9*^	2.6 ± 1.0	0.003
GV (cm/yr) (6m)	7.1 (5.7, 8.5)	7.0 (5.6, 9.0)	10.2 (8.1, 13.8)*#	0.007
BA-CA (yrs) (1yr)	2.3 (1.7, 2.8)	2.2 (1.7, 2.9)	2.9 (0.3, 4.8)	0.883
Height-SDS for bone age (1yr)	-1.5 (-1.9, -1.0)	-1.4 (-1.7, -1.0)	-0.4 (-1.9, 1.7)	0.097
ΔHeight-SDS for bone age (1yr)	0.3 (0.0, 0.5)	0.3 (0.1, 0.5)	0.0 (-0.3, 0.8)	0.575

*Significant difference vs. No lesion; # Significant difference vs. Other lesions; ^ Significant difference vs. Pathogenic lesion; m=months; yr=year.

Normally distributed data are presented as mean ± SD; non-normally distributed data are presented as median (P25, P75).

**Table 7:** Clinical characteristics of boys with CPP and EFP after GnRHa treatment (N = 71).

Characteristic	MRI (-)	Other lesions	P-value
Testicular volume (ml) (6m)	12.8 ± 5.0	15.3 ± 5.9	0.157
BA-CA (yrs)	2.3 ± 0.8	2.3 ± 0.8	0.954
Height-SDS for bone age	-1.8 ± 0.7	-1.6 ± 0.5	0.272
cumulative 6-month therapeutic dose (μg/kg)	588.1 (517.3, 664.1)	556.0 (507.3, 659.2)	0.473
Basal LH (IU/L) (3-6m)	0.4 (0.2, 0.5)	0.3 (0.2, 0.4)	0.685
Peak LH (IU/L) (3-6m)	1.0 (0.6, 1.5)	0.7 (0.5, 1.1)	0.180
Testicular volume (ml) (1yr)	9.9 ± 3.8	10.9 ± 3.9	0.480
GV (cm/yr) (6m)	7.6 (6.9, 9.9)	9.4 (8.0, 10.5)	0.142
BA-CA (yrs) (1yr)	1.6 ± 2.4	2.2 ± 1.0	0.516
Height-SDS for bone age (1yr)	-1.3 ± 0.7	-0.9±-0.5	0.096
ΔHeight-SDS for bone age (1yr)	0.3 ± 0.4	0.5 ± 0.3	0.272

*Significant difference vs. No lesion; # Significant difference vs. Other lesions; ^ Significant difference vs. Pathogenic lesion; m=months; yr=year.

Normally distributed data are presented as mean ± SD; non-normally distributed data are presented as median (P25, P75).

## 4 Discussion

This study analyzed brain MRI findings in 1,087 children with central precocious puberty (CPP) and early and fast puberty (EFP) to investigate the prevalence of intracranial lesions and their impact on pubertal progression and response to gonadotropin-releasing hormone agonist (GnRHa) therapy. Our key findings are as follows: First, intracranial lesions, particularly pathogenic ones, are highly concentrated in younger children with CPP. Second, the detection rate of pathogenic lesions is extremely low in older children with CPP and in all children with EFP. Third, incidental structural changes such as Rathke’s cleft cysts, pineal cysts, and pituitary microadenomas show no difference in clinical characteristics or therapeutic response compared to children without lesions. Finally, CPP associated with hypothalamic hamartomas or gliomas requires higher doses of GnRHa for effective management.

### 4.1 Prevalence and spectrum of intracranial lesions in children with CPP and EFP

Intracranial lesions were identified in 14.5% of girls and 19.6% of boys with CPP or EFP, while definitive pathogenic lesions were observed in only 2.1% and 3.9%, respectively. Notably, there was no statistically significant difference in the total detection rate between girls and boys. Although the proportion of pathogenic lesions was slightly higher in boys (3.9%) than in girls (2.1%), it was far lower than the traditionally cited range of 50–70% (10). This finding aligns with trends observed in recent domestic and international studies. For instance, Ayfer et al. (7) reported that only 26% of boys with CPP had an organic etiology, predominantly in those under 7 years of age. Similarly, studies by Jong et al. (8) and the team of Junfen Fu in China (6) indicated that the proportion of organic CPP in boys ranged from only 7% to 16.3%. While the predominance of idiopathic etiology in girls with CPP is well established, our findings challenge the traditional view in boys and confirm that idiopathic causes remain dominant across both sexes in the Chinese population.

The prevalence of intracranial lesions demonstrated a significant age-dependent trend. In our cohort, girls under 6 years and boys under 7 years exhibited significantly higher detection rates of intracranial lesions—particularly pathogenic ones (33.7% and 53.8%, respectively)—compared to their older counterparts. This suggests that young age is the most critical risk factor for organic intracranial lesions (5).

Crucially, all pathogenic lesions (hypothalamic hamartomas and gliomas) were identified exclusively in children with CPP; none were detected in the EFP group. Furthermore, the detection rate of pathogenic lesions dropped to negligible levels (0.2%-0.6%) in girls aged ≥6 years and boys aged ≥7 years with CPP. In line with these observations, very recently published Clinical Practice Guidelines on Precocious Puberty (Latronico AC et al., J Clin Endocrinol Metab 2026) have clearly suggested that brain MRI should not be routinely performed in girls aged 6-8 years and boys aged 8-9 years. Our data are in perfect line with these recommendations (11). These findings provide a critical evidence base for optimizing clinical imaging screening strategies. Historically, brain/sellar region MRI has been widely employed for etiological screening in CPP and EFP, yet its necessity, particularly in older, asymptomatic children, remains controversial (12–14). Our results suggest that for EFP children without neurological symptoms, as well as for girls with CPP aged ≥6 years and boys with CPP aged ≥7 years, the clinical benefit of routine MRI screening at initial diagnosis (in terms of detecting pathogenic lesions requiring urgent intervention) may be limited. Consequently, screening intensity in these subgroups could be appropriately reduced to avoid overutilization of medical resources, alleviate financial burdens on families, and minimize potential risks to children associated with the examination process (e.g., sedation) (15, 16).

### 4.2 Impact of different intracranial lesion types on pubertal progression and GnRHa efficacy

This study provides the first systematic comparison of clinical characteristics and therapeutic responses among three groups of children: those with no intracranial lesions, those with incidental structural lesions (“other lesions”), and those with established pathogenic lesions. Our results demonstrate that children with CPP associated with hypothalamic hamartomas or gliomas exhibit a significantly earlier age at pubertal onset (mean 1.9 years for girls and 1.0 years for boys) compared to the other two groups, this finding aligns with the characteristic of earlier disease onset reported in previous studies (17). Furthermore, these patients present with a higher degree of gonadal axis activation at diagnosis, evidenced by elevated basal and stimulated LH levels. These findings align with the theoretical framework suggesting that hypothalamic hamartomas can act as ectopic GnRH pulse generators or interfere with normal inhibitory pathways via mechanisms such as TGF-α secretion, thereby driving premature puberty (18–21). However, several limitations must be acknowledged. Notably, no male patients with pathogenic lesions had post-treatment data available, precluding a therapeutic outcome analysis for this subgroup. Furthermore, the number of male patients with incidental findings (n = 7) and girls with pathogenic lesions (n = 9) were both limited, which restricts the statistical robustness and generalizability of our sex-specific conclusions. Therefore, our proposal for age-stratified MRI screening in boys should be interpreted with caution and requires validation in larger, multi-center cohorts.

Regarding treatment, this study offers direct clinical evidence: the pathogenic lesion group required significantly higher cumulative doses of GnRHa (median 852.0 μg/kg over the first 6 months in girls) to achieve gonadal suppression. Additionally, their linear growth velocity (GV) remained faster than that of the other groups even after six months of treatment. This corroborates the notion that axis activation driven by such lesions is more refractory and poses greater clinical challenges (22). However, on a positive note, once dosages were adjusted to sufficient levels, the degree of sex hormone suppression (basal and peak stimulated LH) achieved at 3-6 months in these patients was comparable to that in the no-lesion group treated with standard doses. This indicates that GnRHa therapy remains effective for this subgroup through individualized dose optimization.

Moreover, a key conclusion is that most detected abnormalities in this female CPP cohort—specifically Rathke’s cleft cysts (7.7%), pineal cysts (2.2%), non-suprasellar arachnoid cysts, pituitary microadenomas, and pituitary stalk thickening—appear to be incidental findings. Evidence supporting this includes (1): these lesions were distributed similarly between EFP and CPP groups; (2) children harboring these lesions showed no differences in age at pubertal onset, bone age progression, or baseline hormone levels compared to those with no lesions; and (3) during GnRHa therapy, their required dosages, hormonal suppression levels, growth velocities, and improvements in height SDS were identical to the no-lesion group. These results are consistent with the perspectives of Pedicelli et al. (4) and Chiu et al. (23), who posited that such imaging findings are more likely concomitant phenomena rather than etiologies. With respect to pituitary stalk thickening, although it can be a marker for proliferative disorders such as germinoma, our comprehensive workup in this cohort did not reveal any malignant features. All patients with this finding exhibited normal hypothalamic-pituitary function and had negative serum tumor markers (alpha-fetoprotein and human chorionic gonadotropin), with no clinical evidence of Langerhans cell histiocytosis. Consistent with other incidental findings, this subgroup showed no alteration in disease progression or therapeutic response, suggesting that isolated stalk thickening without systemic signs may not necessitate aggressive intervention. Notably, regarding pineal cysts, our findings differ from those of Wei Haiyan et al. (24), who suggested they might accelerate pubertal progression; this discrepancy may stem from our cohort comprising smaller cysts (all < 5 mm in maximum diameter) that were all incidentally detected. By comparison, over half of the cysts reported in their study exceeded 5 mm in size. Therefore, for children with CPP or EFP found to have only such lesions, there is no need to alter standard GnRHa treatment strategies, nor is there a justification for excessive anxiety or unnecessary repeat imaging.

## 5 Conclusions

1. Younger children with CPP (girls <6 years, boys <7 years) exhibit a high detection rate of intracranial lesions, particularly pathogenic tumors; routine brain/sellar region MRI screening is recommended for this subgroup.
2. For older children with CPP (girls ≥6 years, boys ≥7 years) and all children with EFP, the detection rate of pathogenic intracranial lesions is low. The necessity of routine MRI screening should be re-evaluated in these populations to avoid excessive examination.
3. Intracranial structural abnormalities such as Rathke’s cleft cysts and pineal cysts, as well as pituitary microadenomas, likely represent incidental findings in CPP/EFP that do not influence pubertal progression; favorable therapeutic outcomes can be achieved with standard GnRHa dosing.
4. Children with CPP associated with hypothalamic hamartomas or gliomas present with earlier pubertal onset and more profound activation of the gonadal axis, typically requiring higher doses of GnRHa for effective management.

## 6 Innovations and limitations

### 6.1 Innovations

This study provides large-scale epidemiological data on intracranial lesions in Chinese children with CPP and EFP. Uniquely, it offers the first systematic analysis of how different lesion types specifically impact the efficacy of GnRHa therapy, thereby furnishing direct evidence to guide clinical decision-making.

### 6.2 Limitations

The study is constrained by its single-center retrospective design. Additionally, the sample size for certain subgroups was relatively small, and the follow-up duration for GnRHa therapy was limited to one year; thus, long-term therapeutic efficacy warrants further observation.

## Data Availability

The raw data supporting the conclusions of this article will be made available by the authors, without undue reservation.
